# Acute Limb Ischemia in the Young: A Rare Case of Essential Thrombocytosis

**DOI:** 10.1155/2021/2563578

**Published:** 2021-11-20

**Authors:** Johanes Nugroho, Ruth Gunadi

**Affiliations:** ^1^Department of Cardiology and Vascular Medicine, Faculty of Medicine, Universitas Airlangga, Surabaya, Indonesia; ^2^Dr. Soetomo General Hospital, Surabaya, Indonesia

## Abstract

Acute limb ischemia (ALI) is rarely observed in young populations. The hypercoagulable state is a notable cause of ALI other than artery disease progression and cardiac embolization. A hypercoagulable state occurs in essential thrombocytosis because of the overproduction of hematopoietic cells secondary to the mutation of the JAK2, CALR, or MPL genes. We report a rare case of a 37-year-old woman presenting with Rutherford IIA ALI in the left lower extremity. Laboratory data revealed she had a platelet count reaching up to 1.38 mil/*μ*L, with other blood profiles being normal. A JAK2 mutation examination was later performed and proved positive. After careful management with catheter-directed thrombolysis, surgical thrombectomy, and cytoreductive therapy using hydroxyurea, the symptoms subsided and eventually restored the patient to physical activity in less than one month.

## 1. Introduction

Patients with peripheral arterial disease (PAD) are at risk for ischemic events, which can cause significant morbidity and reduce functional status and the quality of life. Acute limb ischemia (ALI), which results from a sudden decrease in limb perfusion, can cause tissue death and threaten limb viability [1]. Sudden decreased perfusion can be caused either by progressive arterial disease, cardiac embolization, hypercoagulable disease, or complications related to vascular procedures [2]. A part of the hypercoagulation spectrum is essential thrombocytosis (ET). The incidence of ET increases with age, with most patients presenting between the ages of 50 and 60 years [3]. There have been previous case reports of cardiovascular involvement with ET in the fourth decade but not ALI with ET [4]. Hence, a young woman presenting with a complication of ET is considered rare.

## 2. Case Presentation

**Figure 1:**
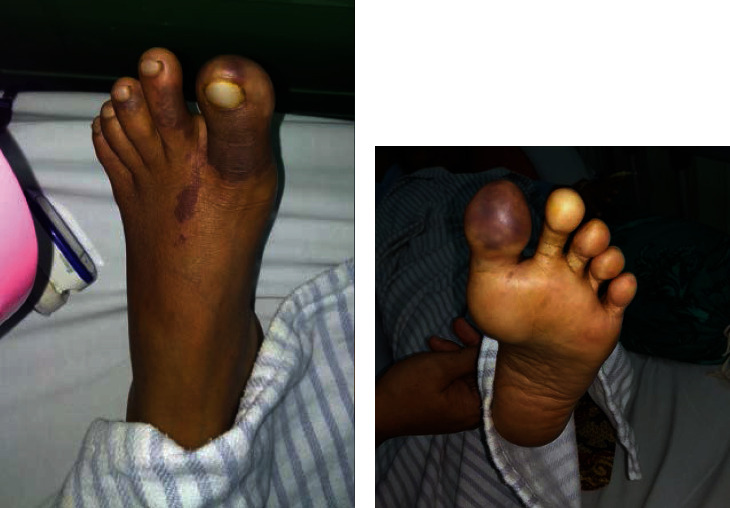
Image of dorsal (a) and ventral (b) views of the left foot in the initial settings with bluish pale area mostly distributed in digits I and II.

A 37-year-old female patient presented with a chief complaint of pain in the toes of her left foot since approximately 1-week prior to hospitalization. She complained of persistent pain which was aggravated with mobilization. In addition, her left toes often felt numb accompanied by a tingling sensation, with a bluish and pale colorization. She denied any current use of medication or prior hospitalizations, and her past medical history was otherwise unremarkable. She had two biological children, both born per vaginam. On physical examination, she had tachycardia with an initial heart rate of 102 beats per minute. Left inferior extremity assessment revealed a weakened pulsation in her left femoral artery, an absence of distal pulsations, and dry, cold, and pale digits (first to fifth) (Figure 1). Motoric abilities were unimpaired, but sensory impairment (numbness) was observed in her left foot.

Electrocardiogram, chest radiograph, and transthoracic echocardiography revealed no abnormality or intracardiac thrombus. Doppler ultrasound of the lower extremity arteries revealed triphasic waves in the right superficial femoral artery. For the left inferior extremity, a thrombus was documented in the common femoral artery (CFA); hence, arterial flow from the CFA towards the distal end was difficult to evaluate.

The patient's initial platelet count was 812 × 103/*μ*L, while other routine blood profiles were within normal limits. A blood smear test showed thrombocytosis with giant platelets, leukocytosis, and immature granulocytes. Subsequently, a JAK-2 mutase examination was performed based on the suspicion of ET.

**Figure 2:**
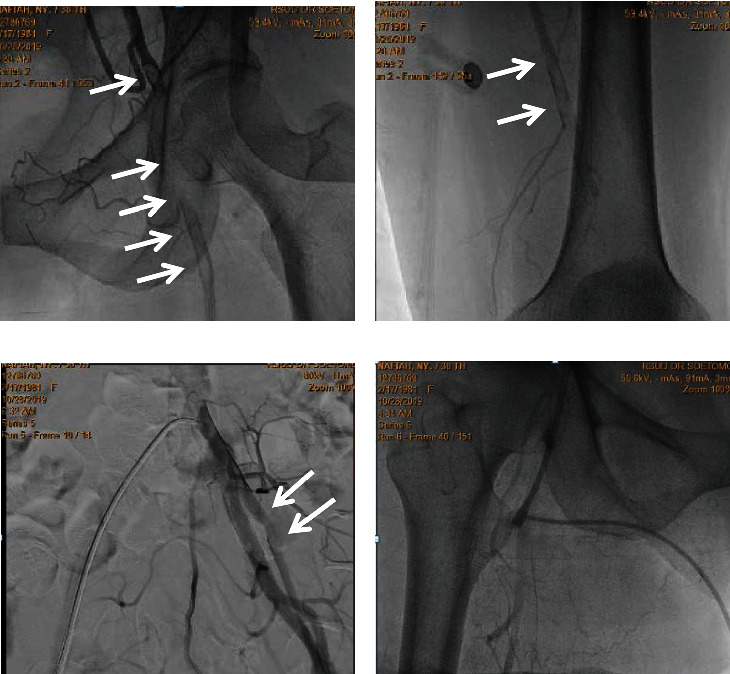
Arteriographic findings of the inferior extremities show that diffuse thrombus was visualized in the left common femoral artery (a) continuing until the superficial femoral artery and distal femoral artery (b). A thrombus appears on left external iliac artery (c). Contrast flow is normal in the right inferior limb (d).

She then underwent an arteriography (Figure 2) with catheter-directed thrombolysis (CDT). A 6-French pigtail catheter was inserted through the right femoral artery and directed towards the left femoral artery. Digital subtraction cineangiography was performed using an injection of 10 mL of contrast with an anteroposterior projection. Arteriography revealed a thrombus that was as high as the left distal CFA. A total of 100,000 units of Fibrion (Streptokinase) was injected into the left femoral artery. No complications occurred during or after the procedure.

After the procedure, the patient was admitted to the intensive cardiac care unit and administered 100,000 units/hour of streptokinase via CDT, with hourly clinical evaluation and monitoring. At clinical evaluation 2 hours postarteriography, she complained of severe pain in her left lower leg. Accordingly, the patient was scheduled for emergency surgical thrombectomy. Antegrade and retrograde surgical thrombectomy of the posterior tibial artery was performed, with results of restored flow of the left popliteal artery and posterior tibial artery. During inpatient treatment, the platelet count increased drastically (from 812 × 103/*μ*L to 1.380 × 103/*μ*L).

**Figure 3:**
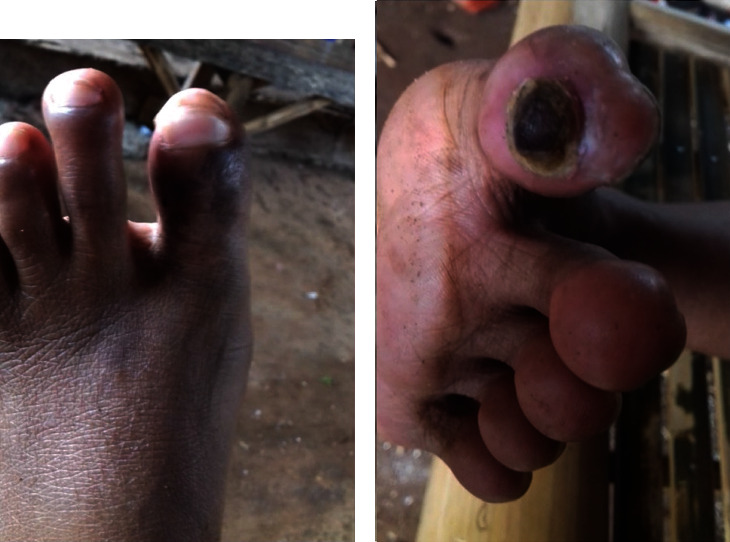
Image of dorsal (a) and ventral (b) views of the left foot in the six-month follow-up, with significant clearing of cyanotic area.

Once the diagnosis of ET was established, medical therapy was started immediately with hydroxyurea and clopidogrel once daily at doses of 1,000 mg and 75 mg, respectively. At the one-month follow-up, her platelet count had decreased by half to 434 × 103/*μ*L with improved clinical results. At the six-month follow-up, a marked reduction in cyanotic areas was observed throughout the first and second digits, with improved mobility and daily performance (Figure 3).

## 3. Discussion

Along with revascularization, etiological evaluation of ALI has become a priority, especially if the patient belongs to a young demographic, considering that the incidence of ALI occurs mostly in the elderly with various comorbidities. In the present case, the patient was a young woman who had no prior history of comorbidities; thus, an event such as ALI warrants considerations of etiologies beyond the more common ones.

Progressive arterial disease deemed unlikely due to her young age and no intracardiac thrombus on echocardiography. Hence, differential diagnoses include hypercoagulable states and myeloproliferative (MPN) disorders, which can manifest in thrombosis and symptoms of ALI in the young population. Since our patient's hematology data suggested high platelet levels in three separate occasions, with other hematology profile to be normal, our suspicion was focused on MPN disorders. According to recent observational studies, there were three common MPN disorders in the young: polycythemia vera (PV), essential thrombocytosis (ET), and primary myelofibrosis (PMF) [5, 6]. Since our patient's Hb and Hct levels were normal, PV became less suspicious. In PMF, although sharing a common JAK2, CALR, or MPL mutation, anemia is typically present and increases over time accompanied by mild to moderate leukocytosis, mild to marked thrombocytosis, abnormal cytokine expression, cachexia, and hepatosplenomegaly [7, 8]. With clinical signs and symptoms suggestive of ET, supported by positive JAK2 V617F mutation, the diagnosis of ET was established.

The incidence of ET varies from 1.0 to 2.5 per 100.000 individuals/year, with a prevalence of 38–57 per 100,000 individuals reported between 2008 and 2010, mostly among women [3, 9]. According to the World Health Organization, ET is a disease that occurs when the platelet count is more than 450 × 103/*μ*L with proven mutations in Janus kinase 2 (JAK2) and calreticulin (CALR), or mutations in the oncogene leukemia myeloproliferative oncogene (MPL) virus, with exclusion of clonal disease or reactive causes [10]. Approximately 50% of patients with ET have JAK2 mutations [3].

Thrombosis is the most common cause of mortality and morbidity in patients with ET. The incidence of thrombosis is approximately 20%, followed by the incidence of bleeding (10%), and the estimated incidence of conversion (from thrombosis to hemorrhage) is less than 1% [9, 11]. In general, the management of ET includes control of cardiovascular risk factors and cytoreductive therapy, for example, hydroxyurea, in patients who are at high risk of thrombotic events [12]. Treatment with low-dose acetylsalicylic acid (aspirin) is crucial for the prevention of primary and secondary thrombosis in this patient group [13]. However, in patients with ALI, single antiplatelet therapy (SAPT) with clopidogrel is the preferred choice [2]. Hydroxyurea 500–1000 mg once daily is the first line of choice in most patients because of its effective thrombotic reduction profile with minimal side effects [14]. The aim of the treatment is to maintain a platelet count of <400 × 103/*μ*L [13, 14]. Although the patient in the present case achieved a platelet count as low as 434 × 103/*μ*L, the therapy target was not achieved due to poor regular intake of hydroxyurea, as the patient lived in a rural area and had limited access to cytoreductive drugs.

Long-term anticoagulant therapy may be combined with SAPT when a revascularization procedure is performed. Treatment using anticoagulants and SAPT can be considered for more than 1 month in patients with high ischemic risk or when there are other strong indications for long-term SAPT [2]. ET combined with ALI is classified in the high ischemic risk group; thus, it is advisable to administer SAPT and oral anticoagulants for >1 month, and doses should be monitored with a target International Normalized Ratio of 2.0–2.5 [2, 15]. Thus, our patient had increased mobile activity in less than two months after receiving proper revascularization and medication. ET is an indolent disease with a good prognosis. The reported life expectancy is 33 years, and in younger patients, it is 60 years [16]. Although indolent, ET patients have poorer life expectancy than the general population due to thrombotic events that may complicate the disease [17].

In cases of ALI associated with neurological deficits, immediate revascularization is mandatory; investigations and imaging should not delay patient revascularization interventions [13]. Various revascularization modalities can be applied, including percutaneous CDT, percutaneous mechanical thrombus extraction or thrombus aspiration (with or without thrombolytic therapy), and surgical thrombectomy, bypass, and/or arterial repair. The choice of a revascularization strategy will depend on the presence of a neurologic deficit, duration of ischemia, localization, comorbidity, type of blood vessel (artery or graft), and therapy-related risk and outcome. Because of favorable morbidity and mortality rates, endovascular therapy is often preferred by clinicians, especially in patients with severe comorbidities [1, 18].

The modern concept of combined intraarterial thrombolysis and catheter-based clot removal can achieve an amputation rate of <10% within 6 months [1]. In the present case, due to a moderate neurological deficit, percutaneous CDT was performed immediately as soon as the ALI diagnosis was established. However, during follow-up, the patient's condition did not improve and prompted surgical thrombectomy. Subsequently, combined with adequate antithrombotic and hydroxyurea, her clinical condition and performance allowed her to be discharged after 10 days of hospitalization.

## 4. Conclusion

Cases of acute limb ischemia (ALI) in the young population are rare and should trigger investigations to determine the underlying cause. A frequently overlooked etiology of ALI is hypercoagulability, including essential thrombocytosis, which may cause extreme thrombocytosis, bleeding, or progressive arterial damage due to erythromelalgia. Revascularization interventions and medical therapy must be performed according to clinical protocols and indications. Long-term cytoreductive therapy with hydroxyurea is usually sufficient to control platelet count and prevent complications of thrombocytosis in patients with essential thrombocytosis.

## Data Availability

The data supporting this case report are from previously reported studies and datasets, which have been cited.
